# A polynomial based model for cell fate prediction in human diseases

**DOI:** 10.1186/s12918-017-0502-5

**Published:** 2017-12-21

**Authors:** Lichun Ma, Jie Zheng

**Affiliations:** 1 Biomedical Informatics Lab, School of Computer Science and Engineering, Nanyang Technological University, Singapore, 639798 Singapore; 2 Genome Institute of Singapore, A*STAR, Singapore, 138672 Singapore; 3 Complexity Institute, Nanyang Technological University, Singapore, 637723 Singapore

**Keywords:** Cell fate prediction, Cell death, Mathematical modeling, Polynomial, Apoptosis pathway, Correlation analysis, Single-cell gene expression

## Abstract

**Background:**

Cell fate regulation directly affects tissue homeostasis and human health. Research on cell fate decision sheds light on key regulators, facilitates understanding the mechanisms, and suggests novel strategies to treat human diseases that are related to abnormal cell development.

**Results:**

In this study, we proposed a polynomial based model to predict cell fate. This model was derived from Taylor series. As a case study, gene expression data of pancreatic cells were adopted to test and verify the model. As numerous features (genes) are available, we employed two kinds of feature selection methods, i.e. correlation based and apoptosis pathway based. Then polynomials of different degrees were used to refine the cell fate prediction function. 10-fold cross-validation was carried out to evaluate the performance of our model. In addition, we analyzed the stability of the resultant cell fate prediction model by evaluating the ranges of the parameters, as well as assessing the variances of the predicted values at randomly selected points. Results show that, within both the two considered gene selection methods, the prediction accuracies of polynomials of different degrees show little differences. Interestingly, the linear polynomial (degree 1 polynomial) is more stable than others. When comparing the linear polynomials based on the two gene selection methods, it shows that although the accuracy of the linear polynomial that uses correlation analysis outcomes is a little higher (achieves 86.62%), the one within genes of the apoptosis pathway is much more stable.

**Conclusions:**

Considering both the prediction accuracy and the stability of polynomial models of different degrees, the linear model is a preferred choice for cell fate prediction with gene expression data of pancreatic cells. The presented cell fate prediction model can be extended to other cells, which may be important for basic research as well as clinical study of cell development related diseases.

## Background

Many human diseases are caused by over proliferation or progressive death of specific cells [1, 2]. One notorious example that involves uncontrolled cell growth is cancer, which has become a leading killer worldwide [3]. In contrast to abnormal cell growth, excessive cell death also results in serious damage to human body. Abnormal cardiomyocyte death is a hallmark of various cardiovascular diseases (e.g. heart failure) [4, 5]. Neurodegenerative disorders, such as Parkinson’s, Alzheimer’s and Huntington’s diseases underlie the continuous death of specific neurons [6, 7]. Hepatocellular death is an indicator in detecting liver diseases [8]. Pancreatic β-cell deficit is a main character of type 2 diabetes (T2D) [9, 10]. Thus, cell fate has a direct bearing on human health, and the research on cell fate decision facilitates the study of the mechanisms and may pave the way for preventing diseases caused by abnormal cell development.

With the development of biomathematics, mathematical modeling has been employed in formulating hypotheses and interpreting mechanisms of cell fate decision [11–14]. The models can be categorized according to their properties into different groups, e.g. discrete or continuous, static or dynamic, knowledge-driven or data-driven, etc. In addition, different kinds of data, such as gene expression data or protein activity data, are used in these models. Calzone et al. conceived a compact model based on logical formalization of signal transduction for cell fate decision [15]. They also applied a similar model to the study of cancer cell fate determination [16]. This logical model describes the biological processes in a mechanistic way, but it can only perform in a discrete mode and cannot incorporate context-specific information from real data. Tyson et al. constructed a dynamic model (based on ordinary differential equations) to study cell fate of breast cancer cells [17]. Bhattacharya et al. mapped Waddington’s epigenetic landscape to visualize cell fate based on dynamics of gene regulatory network [18]. Both the dynamic model and the Waddington’s epigenetic landscape require the trajectory information of genes (i.e. time-series gene expression data). The aforementioned three models are knowledge-driven models. As advances in experimental techniques to measure biological data and progresses of methods in computer science, data-driven models become very popular in decoding cell fate decision mechanisms. Janes et al. [19] and Lee et al. [20] employed a partial least squares (PLS) regression model to correlate protein activity levels and phenotypic responses of cancer cells. Note that, the PLS regression model is based on linear transformation. Considering the complexity of biological systems (e.g. cross talk and feedback in signaling pathways), the linear model is not convincing to some researchers. Thus, with the utilization of the same dataset in [20], Zhang et al. [21] proposed an exponential model, which performed better in predicting cell fate than the original linear model used in [20]. However, a log transformation to the protein activity data would convert the exponential model into a linear one. Although these models try to study the cell fate decision, the mechanisms remain far from clear.

We intend to build a model to predict cell fate based on single-cell gene expression data, in which a function is employed to demonstrate their relationship. In this work, cell fate is quantified as the probability of cell death. Considering that a function can be represented with Taylor series under certain conditions (i.e. it can be infinitely differentiable at a fixed point), we applied this theory and directly used different degree polynomials to fit the cell fate prediction function. The gene expression dataset was obtained from single-cell transcriptome profiling of human pancreas [22]. Overall, there are 2209 pancreatic cells from patients of T2D and healthy individuals, and a total of 26,179 genes were measured for each cell. As only a small portion of genes are closely related to cell fate decision, a feature (gene) selection step was conducted on the training data. In this study, we used a correlation based feature selection approach, as well as an apoptosis pathway based method. The correlation based method employs Spearman’s correlation analysis approach [23] to conduct gene selection, and the outcomes only depend on the relationship between gene expression data and cell fates (i.e. the polynomial model based on correlation analysis outcomes is a data-driven model). Differently, the apoptosis pathway based method directly regards the genes in the apoptosis pathway as features, and incorporates gene regulation information into the cell fate prediction model (i.e. the polynomial model within genes of the apoptosis pathway is a combination of data-driven and knowledge-driven models, also known as a hybrid model). After the selected genes were obtained, we performed a regression process to refine the cell fate prediction function, and proceeded to the prediction phase. 10-fold cross-validation was carried out to evaluate the performance of our model. Moreover, we analyzed the stability (i.e. discrepancies of the functions when the training data were changed) of the cell fate prediction functions by evaluating the ranges of the parameters as well as computing the variances of the predicted values at randomly selected points. Results show that within both the gene selection methods, linear polynomial performs better than others. When comparing the linear polynomials based on the two gene selection methods, the prediction accuracy of the model based on the correlation analysis outcomes is a little higher (86.62% vs. 84.17%) than the one using genes from the apoptosis pathway. However, the model within genes from the apoptosis pathway is more stable. The proposed polynomial model in our work demonstrates the feasibility of using linear model to predict cell fate. In addition, current data-driven models for cell fate prediction are often assessed by prediction accuracy. The stability analysis in our work provides ways for a comprehensive evaluation of these models.

## Methods

### Polynomial representation of cell fate

When the gene expression profile of a single cell is available, we aim to predict the fate of this cell based on the expression levels of specific genes. To illustrate our model, suppose we are given three cell fate related genes *A*, *B,* and *C*, with the corresponding expression levels of *x*_*A*_, *x*_*B*_, and *x*_*C*_, respectively. Then we build a model to associate the cell fate *P* (*P* ∈ [0, 1]) with the three genes’ expression levels. Suppose that the three genes are independent of each other, then *P* can be represented as:

1 $$ P=f\left({x}_A\right)+g\left({x}_B\right)+h\left({x}_C\right), $$

where *f*, *g*, and *h* are three arbitrary functions. If *f*(*x*_*A*_) is infinitely differentiable at *a* (where *a* is a real or complex number), we can expand *f*(*x*_*A*_) with Taylor series as follows,

2 $$ \sum \limits_{n=0}^{\infty}\frac{f^{(n)}(a)}{n!}{\left({x}_A-a\right)}^n=f(a)+\frac{f^{\prime }(a)}{1!}\left({x}_A-a\right)+\frac{f^{{\prime\prime} }(a)}{2!}{\left({x}_A-a\right)}^2+\cdots . $$

Here, *f* ^(*n*)^(*a*) denotes the *n-*th derivative of *f*(*x*_*A*_) at *a.* Similarly, *g*(*x*_*B*_) and *h*(*x*_*C*_) can be represented with Taylor series respectively. As such, *P* can be rewritten as:

3 $$ {\displaystyle \begin{array}{c}P=\sum \limits_{n=0}^{\infty}\left({k}_{An}{x}_A^n+{k}_{Bn}{x}_B^n+{k}_{Cn}{x}_C^n\right)\\ {}=\sum \limits_{n=1}^{\infty}\left({k}_{An}{x}_A^n+{k}_{Bn}{x}_B^n+{k}_{Cn}{x}_C^n\right)+b,\end{array}} $$

where *k*_*An*_, *k*_*Bn*_ and *k*_*Cn*_ are polynomial coefficients, and *b* is a constant. In some cases, the genes are not mutually independent, e.g., gene *A* promotes the transcription of gene *C*. Then the simultaneous influence of genes *A* and *C* on cell fate *P* is not additive. We employ *f*(*x*_*A*_, *x*_*C*_) to show their synergistic effects. Accordingly, *P* can be represented as:

4 $$ P=f\left({x}_A,{x}_C\right)+g\left({x}_B\right). $$

Similar to the Taylor series representation of a function with one variable (Eq. (2)), we can also expand a function with two variables. If *f*(*x*_*A*_, *x*_*C*_) is infinitely differentiable at a point (*a*, *c*), where *a* and *c* are real or complex values, it can be expressed with Taylor series as follows,

5 $$ {\displaystyle \begin{array}{c}\sum \limits_{n_1=0}^{\infty}\sum \limits_{n_2=0}^{\infty}\frac{{\left({x}_A-a\right)}^{n_1}{\left({x}_C-c\right)}^{n_2}}{n_1!{n}_2!}\left(\frac{\partial^{n_1+{n}_2}f}{\partial {x}_A^{n_1}\partial {x}_C^{n_2}}\right)\left(a,c\right)\\ {}=f\left(a,c\right)+\left({x}_A-a\right){f}_{x_A}\left(a,c\right)+\left({x}_C-c\right){f}_{x_C}\left(a,c\right)\\ {}+\frac{1}{2!}\Big({\left({x}_A-a\right)}^2{f}_{x_A{x}_A}\left(a,c\right)+2\left({x}_A-a\right)\left({x}_C-c\right){f}_{x_A{x}_C}\left(a,c\right)\\ {}+{\left({x}_C-c\right)}^2{f}_{x_C{x}_C}\left(a,c\right)\Big)+\cdots .\end{array}} $$

The subscripts of *f* in Eq. (5) stand for partial derivatives. Considering that *g*(*x*_*B*_) can be represented with Taylor series (similar to Eq. (2)), we can obtain the polynomial representation of *P* by summing up the expansions of *f*(*x*_*A*_, *x*_*C*_)and *g*(*x*_*B*_). Finally, *P* is derived as

6 $$ P=\sum \limits_{n=1}^{\infty}\left[{k}_{An}{x}_A^n+{k}_{Bn}{x}_B^n+{k}_{Cn}{x}_C^n\right]+\sum \limits_{p,q=1}^{\infty }{k}_{AC}^{\prime }{x}_A^p{x}_C^q+b, $$

where *k*_*An*_, *k*_*Bn*_, *k*_*Cn*_ and ${k}_{AC}^{\prime }$ are polynomial coefficients, and *b* is a constant.

The above analysis is based on three genes. Now let us consider *l* genes (*x*_1_, *x*_2_, ⋯, *x*_*l*_) to determine the function of *P*, and assume all the genes are independent of each other. Then *P* can be derived by extending Eq. (3) as follows,

7 $$ P=\sum \limits_{n=1}^{\infty}\left({k}_{1n}{x}_1^n+{k}_{2n}{x}_2^n+\cdots +{k}_{ln}{x}_l^n\right)+b=\sum \limits_{n=1}^{\infty}\sum \limits_{m=1}^l{k}_{mn}{x}_m^n+b. $$

In case of related genes (gene transcription regulation), we can add the cross terms to *P*, i.e.,

8 $$ P=\sum \limits_{n=1}^{\infty}\sum \limits_{m=1}^l{k}_{mn}{x}_m^n+\sum \limits_{i,j}\sum \limits_{p,q=1}^{\infty }{k}_{ij}^{\prime }{x}_i^p{x}_j^q+b, $$

where *x*_*i*_ and *x*_*j*_ represent any two related genes. In the scenario of transcription regulation involving several genes, Taylor series representation of multiple variables can be applied. In practice, we approximate Eqs. (7) and (8) with a finite number of terms. Then, with the utilization of regression methods, the function of *P* can be obtained, when the data of gene expression profiles and cell fates of a group of cells are available.

In this work, polynomials of different degree were employed to fit the function of *P*. The MATLAB function ***regress*** was carried out to conduct the regression process. This function is based on the method of least squares. Detailed information can be found in [24].

### Correlation between cell fate decisions and gene expression profiles

Tens of thousands of genes are encoded in the human genome, and their products play different roles in human body [25]. Specific to cell fate, there are only a portion of genes related to it. Thus, we need to conduct a feature (gene) selection process, in order to find out the cell fate decision related genes. Correlation analysis is a common method for feature selection in machine learning. Therefore, in this study, we employed Spearman’s rank correlation analysis approach [23] to evaluate the relevance between gene expression levels and cell fates. Specifically, for a gene, we computed the Spearman’s rank correlation coefficient between this gene’s expression levels in all the cells and the corresponding cell fates. Spearman’s rank correlation measures the monotonic relationship of two variables. Given two sets of variables ***X*** and ***Y***, the corresponding ranks of the two sets of variables are ***X***_*R*_ and ***Y***_*R*_, respectively. Then the Spearman’s rank correlation coefficient *ρ* is derived by

9 $$ \rho =\frac{\operatorname{cov}\left({\boldsymbol{X}}_R,{\boldsymbol{Y}}_R\right)}{\sigma_{{\boldsymbol{X}}_R}{\sigma}_{{\boldsymbol{Y}}_R}}, $$

where cov(***X***_*R*_, ***Y***_*R*_) denotes the covariance of ***X***_*R*_ and ***Y***_*R*_, ${\sigma}_{{\boldsymbol{X}}_R}$and ${\sigma}_{{\boldsymbol{Y}}_R}$ represent the standard deviations of ***X***_*R*_ and ***Y***_*R*_, respectively. After the correlation coefficient of each gene with cell fates was obtained, we sorted the genes according the absolute values of the coefficients. The highly ranked genes were considered as important for cell fate decision.

### Statistical analysis

Statistical comparison of gene expression levels for two groups of samples (cells from healthy and T2D donors) was carried out by using Student’s *t*-test. The difference between the two groups was considered as significant if the *p*-value is less than 0.05.

## Results

### Single-cell gene expression in pancreas

**Fig. 1:**
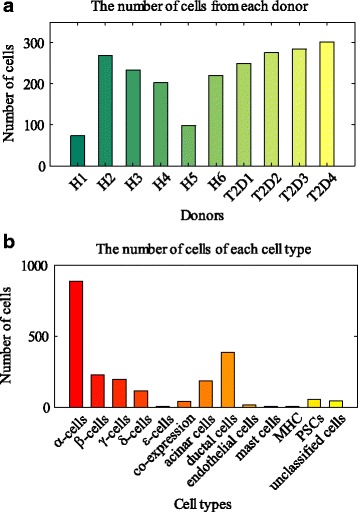
Experiment data of human pancreatic cells. **a** Numbers of cells obtained from 10 donors. H1~H6 represent six healthy donors, while T2D1~T2D4 denote four T2D donors. **b** Distribution of the cells among of cell type. There are 12 types of cells, including α-cells, β-cells, γ-cells, δ-cells, ε-cells, co-expression cells, acinar cells, ductal cells, endothelial cells, mast cells, major histocompatibility complex (MHC) class II cells, and pancreatic stellate cells (PSCs). Others are grouped into unclassified cells

The single-cell gene expression dataset was obtained from [22]. This dataset comprises profiles of totally 2209 pancreatic cells, belonging to 10 donors. Among the donors, six were healthy while four experienced T2D. The numbers of cells obtained from the donors are shown in Fig. 1a. The human pancreas is composed of exocrine and endocrine regions, corresponding to exocrine cells and endocrine islets. Most of the exocrine cells are acinar cells or ductal cells, which play an important role in digestion by secreting and transporting digestive enzymes [26]. Endocrine islets mainly contain hormone-producing cells, e.g. α-cells, β-cells, γ-cells, δ-cells, and ε-cells [27]. Figure 1b shows the distribution of the pancreatic cells among cell types.

**Fig. 2:**
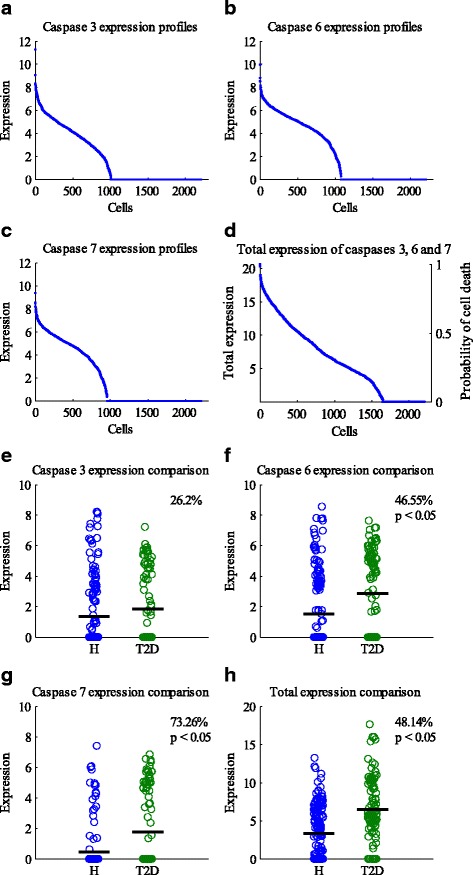
Gene expression profiles of cell death executioner enzymes. **a**-**c** Gene expression profiles of caspases 3, 6, and 7 of all 2209 the cells. The cells are sorted according to the expression levels in descending order. **d** Combined gene expression (caspases 3, 6 and 7) and cell death probabilities of cells. The data are sorted according to the total gene expression levels. **e**-**h** Comparison of expression levels of caspases 3, 6, 7, as well as the total expression levels (caspases 3, 6 and 7) in β-cells. Overall, there are 270 β-cells. Among the cells, 171 of them are from healthy donors, and the rest from T2D donors. The expression of caspases 6, 7 and the total expression of the healthy group (represented with H in the figures) are all significantly lower than that of the T2D group. Only the expression levels of caspase 3 of the two groups do not show significant difference. The bold dark lines indicate the mean values of each group. The extent of differences is provided in the each figure. It is calculated by 1−MTCH/MTCT2D, where MTCH and MTCT2D are the mean values of the total expression levels of caspases 3, 6, and 7 of the healthy and T2D groups, respectively

In this study, we intend to predict the fate of a cell according to its gene expression data. Specifically, within its gene expression profiles, we try to predict the probability of cell death (which is represented by apoptosis in this paper). It is well known that caspases 3, 6, and 7 are executioner enzymes in apoptosis. Thus, it is reasonable to use their expression levels as markers for measuring the cell death probability. Figure 2a-c present the expression levels of caspases 3, 6, and 7 in all the 2209 cells in descending order. We employed the combined expression levels of caspases 3, 6, and 7 to measure the likelihood of cell death. Then the death probability of a cell can be derived by dividing its combined expression level of caspases 3, 6, and 7 by the maximum total expression value of the 3 caspases in all the cells (Fig. 2d). In this work, a cell is considered as less likely to die if the death probability is less than 0.5; otherwise, it is assumed to die with a high probability.

To further verify the feasibility of adopting the total expression of caspases 3, 6, and 7 as a measure of the chance of cell death, we compared the expression of these genes in β-cells between healthy donors and T2D donors (Figs. 2e~2h). β-cells control the secretion of insulin, which can maintain the homeostasis of glucose in blood. Several studies have shown that β-cell deficit ranges from about 20% to 65% in patients with T2D [28, 29]. Student’s *t*-test was performed to show the differences in caspase expression levels between the two groups, with *p*-value <0.05 taken as statistically significant. As shown in Figs. 2f~2h, the expression levels of caspase 6, 7, and the total caspase expression levels (caspases 3, 6, and 7) in β-cells of the healthy group and the T2D group are significantly different. In addition, the individual and total expression levels of caspases 3, 6, and 7 of the healthy group are lower than those of the T2D group. Thus, the β-cells of T2D donors are more vulnerable to cell death compared with the ones of the healthy donors. This is consistent with the β-cell deficit facts in T2D. Thus, it is rational to use the total expression level of caspases 3, 6, and 7 in a cell to measure the likelihood of cell death.

### Cell fate prediction based on correlation analysis

Single-cell gene expression data are available for 2209 cells. In this study, we adopted 10-fold cross-validation to evaluate the prediction of cell fate (in terms of cell death probability). Thus, the data of cells were randomly divided into ten equal sized subsets of cells. At each time of the cross-validation, nine subsets of data were used for training, and the left one was used for testing. This process was repeated for 10 times (each subset of data is exactly used once as testing data) as one 10-fold cross-validation. Overall, we carried out the 10-fold cross-validation for 10 times, and as such generated 100 simulation results. In other words, we refined a total of 100 cell fate prediction functions, producing 100 prediction results. Then a single estimation of prediction accuracy was obtained by computing the average value of the 100 prediction results. Specific to the prediction accuracy of the tested cells, we compared the predicted cell death probability with the actual one of each cell. If both the values fell in the same interval ([0, 0.5) or [0.5, 1]), it was considered as a correct prediction; otherwise, the prediction was incorrect. Then the prediction accuracy of a testing dataset was derived by dividing the number of cells whose fates are correctly predicted by the total number of tested cells.

**Fig. 3:**
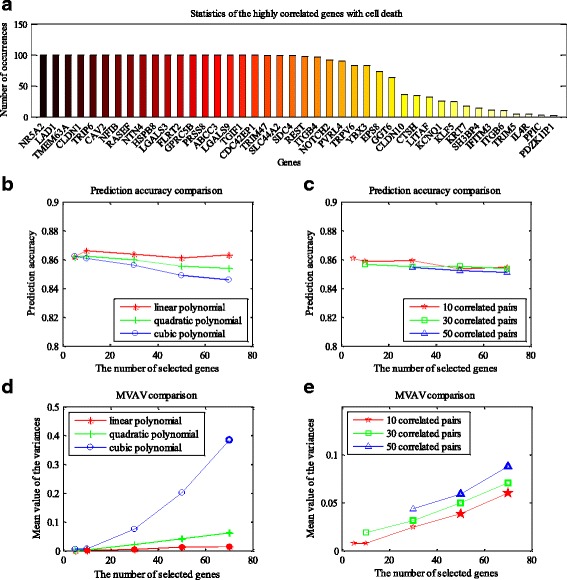
Cell fate prediction based on genes selected by correlation analysis. **a** Highly correlated genes with cell death. These genes were extracted by using Spearman’s rank correlation analysis approach from 100 training datasets (10 times of 10-fold cross-validation), with the top 30 genes highly correlated with cell death selected in each training dataset. **b**, **c** Prediction accuracies of cell death by using different degree polynomial models (linear, quadratic and cubic polynomials) in (**b**), as well as quadratic polynomial model with different number of correlated gene pairs in (**c**). **d**, **e** Stability comparison of different models. For each model, a total of 10,000 randomly selected points were used to measure its stability. After 10 times of 10-fold cross-validation, 100 regression values were obtained for each point. Then the variance of each point can be derived. We use the MVAV (mean value of the 10,000 variances) to assess the instability of each model. Thus, smaller value of MVAV indicates that the model is more stable. The bold markers denote that, within the corresponding model, the 10,000 variances obey gamma distribution

For each of the 2209 cells, the expression levels of 26,179 genes were measured. Thus, except for the marker genes (caspases 3, 6 and 7), a total of 26,176 genes can be used to conduct cell death prediction. However, not all of these genes are closely related to cell death. Hence, we first carried out a feature (gene) selection process to the cells used for training. Spearman’s rank correlation analysis was employed to extract genes that were highly related to cell death. Then, these genes were used as features of the training samples to refine the cell fate prediction function. We also extracted the corresponding genes of the testing cells, in order to make prediction. Figure 3a provides an example of the top 30 genes correlated with cell death from each training dataset. As the data used for training are different in each simulation, the derived top 30 genes may vary slightly among the training datasets. After conducting the 10-fold cross-validation for 10 times (100 times of training), 42 genes were extracted. Among them, 18 genes occur 100 times, i.e. they are repeatedly selected in the top 30 highly correlated genes from all the 100 training datasets. Several genes correlated to cell death are evidently related to apoptosis. For example, in chronic myeloid leukemia progenitor cells, RASEF was shown to induce apoptosis by activating caspases 3 and 9 [30]. Smith et al. reported that HSPB8 inhibits tumor growth by activating apoptosis pathways [31]. Zhang et al. demonstrated that PRSS8 promotes apoptosis and suppresses tumor growth in hepatocellular carcinoma [32]. In addition, evidence for the roles of LGALS9, LITAF and SH3BP4 in apoptosis has also been shown in the literature [33–35]. Except for these genes that are directly related to apoptosis, other genes may be involved in cell growth or other cellular processes. In fact, the functions of many genes and their roles in cellular processes are still not well known. Thus, not only does the correlation based method extract cell fate decision related genes, but it also provides clues for the genes’ functions if they are not completely understood.

We employed different degree polynomial models (linear, quadratic and cubic polynomials) to predict the cell fate. The function ***regress*** in MATLAB was called to conduct the regression analysis. We selected 5, 10, 30, 50, and 70 cell death related genes (according to the absolute values of Spearman’s correlation coefficients) from a training dataset. The prediction results are shown in Table 1 and Fig. 3b. Among the different combinations of models and selected genes, the highest prediction accuracy of 86.62% is achieved by the linear polynomial model on 10 genes. In consideration of gene-gene interactions, we also added cross terms to the quadratic polynomial model. The cross terms were chosen according to the Spearman’s correlation coefficients between gene pairs among the selected genes. We applied the top 10, 30, and 50 pairs of correlated genes in the quadratic polynomial model, respectively. The results are presented in Table 2 and Fig. 3c. Some prediction results are missing when there are too few genes to provide a specified number of gene pairs.

**Table 1:** Cell fate prediction with different degree polynomials. The genes are selected by using correlation analysis approach

Degree of polynomial	Selected genes	5	10	30	50	70
1	Accuracy	0.8620	0.8662	0.8637	0.8612	0.8632
	MVAV	7.7577e-4	0.0013	0.0050	0.0125	0.0146
2	Accuracy	0.8619	0.8623	0.8597	0.8554	0.8538
	MVAV	0.0024	0.0033	0.0216	0.0421	0.0617
3	Accuracy	0.8624	0.8607	0.8561	0.8490	0.8460
	MVAV	0.0055	0.0065	0.0755	0.2020	0.3846

**Table 2:** Cell fate prediction by using quadratic polynomial model with correlated gene pairs

Correlated pairs	Selected genes	5	10	30	50	70
10	Accuracy	0.8608	0.8587	0.8593	0.8535	0.8548
	MVAV	0.0079	0.0080	0.0247	0.0386	0.0602
30	Accuracy	–	0.8566	0.8551	0.8553	0.8537
	MVAV	–	0.0190	0.0315	0.0500	0.0707
50	Accuracy	–	–	0.8545	0.8524	0.8512
	MVAV	–	–	0.0439	0.0592	0.0877

**Fig. 4:**
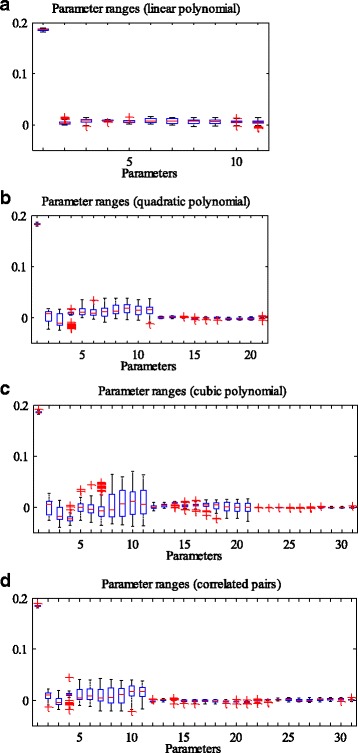
Comparison of regression parameter ranges. The regression parameter ranges of linear (**a**), quadratic (**b**), and cubic polynomial models (**c**), as well as the quadratic polynomial models with 10 correlated gene pairs (**d**). For the linear model, the first parameter represents the constant term of each regression function, and other parameters are arranged according to the importance of the corresponding variables. Each variable stands for the expression level of a gene, and its importance is evaluated by the absolute value of the correlation coefficient with cell death. For the quadratic model, the parameters are arranged in the order of constant term, the parameters of the linear variables, and the parameters of quadratic variables. The ranks of the parameters of both linear and quadratic variables can refer to that of the linear model. The parameters of the cubic model are shown similarly

**Fig. 5:**
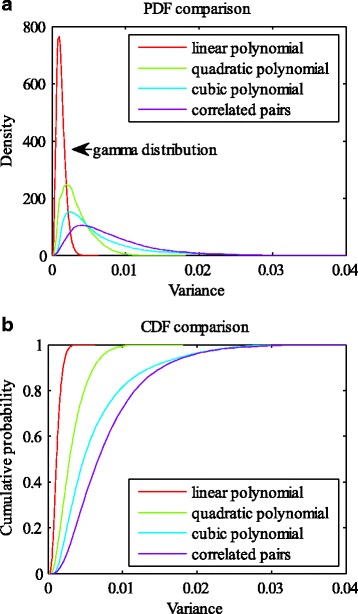
Comparison of PDF and CDF of the models. The PDFs (**a**) and CDFs (**b**) of the variances. For each model (linear, quadratic, cubic polynomials, as well as the quadratic polynomial with ten correlated pairs), we draw the PDF and CDF of the 10,000 variances (as there are 10,000 testing points)

Prediction accuracy is just one performance measure of the models. We also evaluated the stability of different degree polynomial models in our work. Here, stability describes the resistance to changes of the model when different training data are applied. In our simulation, we conducted 10 times of 10-fold cross-validation for each setting of the models and the number of used genes (e.g. 10 genes used in linear polynomial model). Then, for one setting, a total of 100 regression functions were derived as the model was trained with 100 slightly different training datasets. On the one hand, we measured the variation ranges of the regression parameters of the 100 functions. On the other hand, we randomly selected 10,000 points (high dimensional points, with each dimension representing the expression level of one gene), and the regression values of each point can be obtained according to the regression functions. Then 100 regression values were generated for each point, as we simulated 100 times for each setting. Afterwards, the variance of the 100 regression values at each point can be derived. Overall, a total of 10,000 variances were obtained. We can draw the probability density function (PDF) and cumulative density function (CDF) of the variances to examine their distributions. Additionally, the mean value of all the variances (MVAV) could be used to measure the instability of the model, with the smaller value of MVAV denoting high stability. It should be clearly noted that we used the same set of randomly selected points when conducting stability analysis for different models with the same number of genes (e.g. 10 genes applied in both linear and quadratic polynomial models). Figures 4 and 5 present an example of the stability analysis. We adopted 10 genes to refine the linear, quadratic, and cubic polynomial models, as well as the quadratic polynomial model with 10 correlated gene pairs. As shown in Fig. 4, the parameter ranges of the linear polynomial model are the smallest, while the cubic polynomial model is the most volatile. In addition, the PDFs and CDFs show that the distribution of the variances associated with the linear polynomial model is very dense, and the values of the variances are much smaller (Fig. 5). Tables 1 and 2 provide the MVAV for each kind of setting. The linear polynomial model performs better than other order polynomial models (quadratic, cubic polynomials and quadratic polynomial with correlated gene pairs), when the number of applied genes is fixed (Fig. 3d and e).

### Cell fate prediction by using genes in the apoptosis pathway

**Fig. 6:**
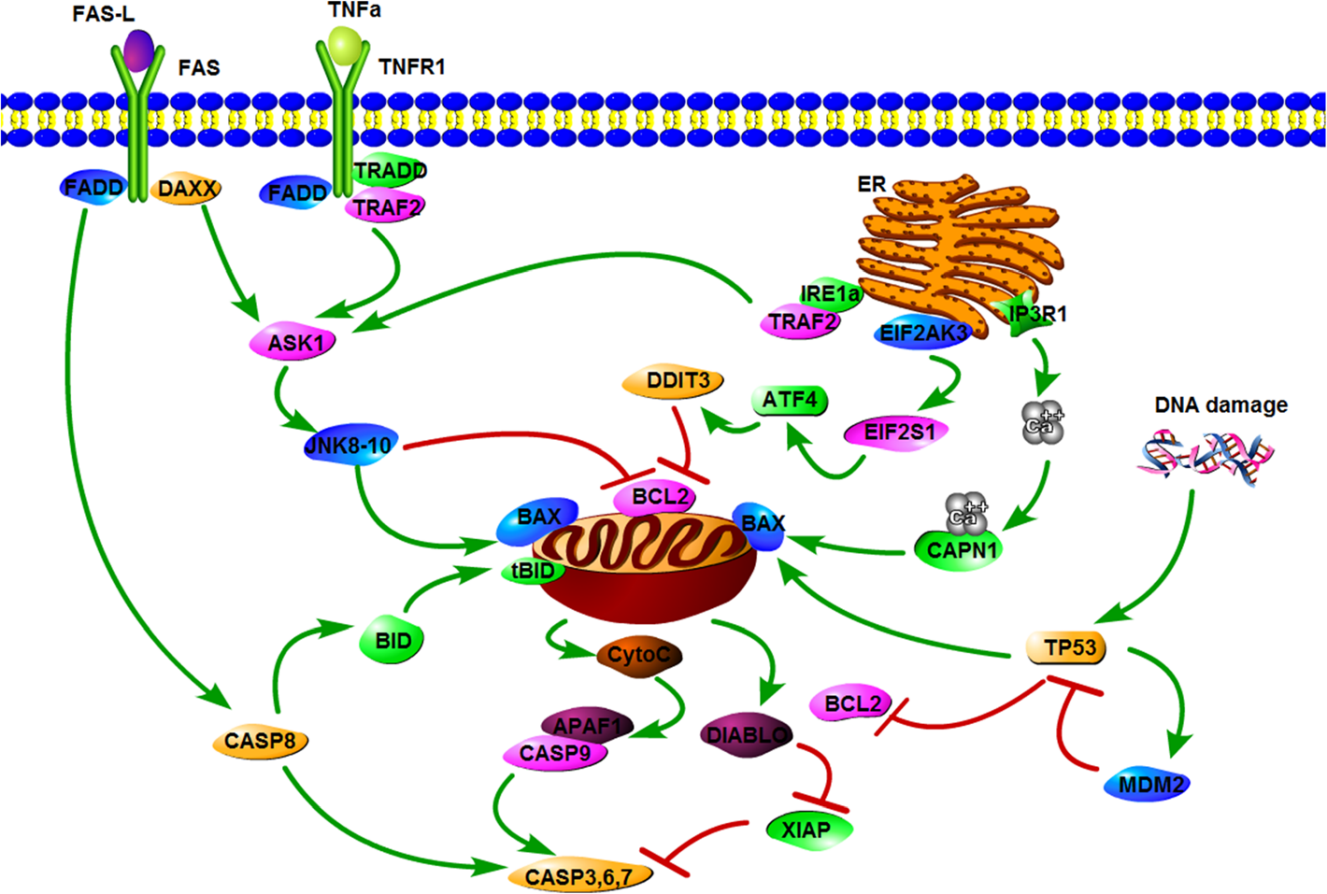
The apoptosis signaling pathway. It mainly involves four parts: the death receptor-induced pathway, endoplasmic reticulum (ER)-mediated pathway, TP53-dependent pathway, as well as the mitochondrial pathway. Stimulated by death ligands (e.g. TNFα), the death receptors recruit adaptor proteins and then activate the mitochondrial pathway or directly induce apoptosis by caspase 8. ER stress promotes apoptosis by ER sensors of IRE1α and EIF2AK3, or release of Ca^2+^ through IP3R1 channel. TP53 plays an important role in apoptosis by activating the transcription of pro-apoptotic proteins (e.g. BAX) while inhibiting the transcription of anti-apoptotic proteins (e.g. BCL2). In mitochondria, activated BAX stimulates the release of cytochrome c, and eventually triggers the caspase cascades

**Fig. 7:**
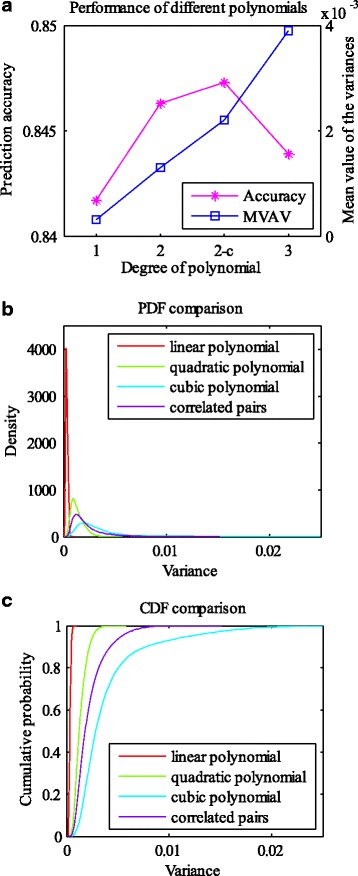
Cell fate prediction by using genes in the apoptosis pathway. **a** The prediction accuracy and the MVAV (mean value of the 10,000 variances) of each model. Smaller MVAV indicates that the model is more stable. 2-c represents quadratic polynomial with correlated gene pairs. **b**, **c** The PDFs and CDFs of the variances. Similar to the results of correlation based approach, there are 10,000 variances for each model

**Fig. 8:**
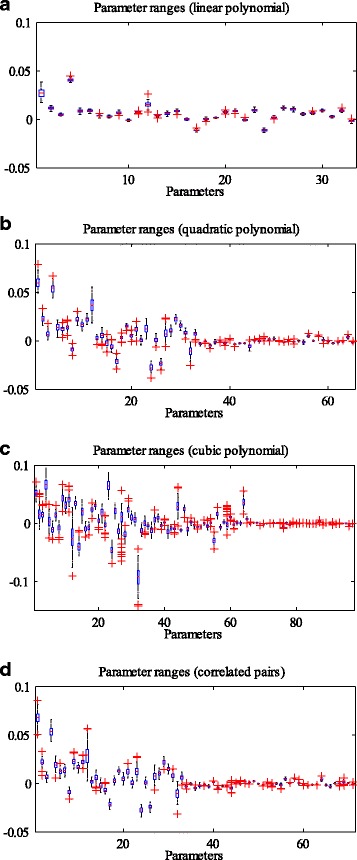
The stability of the models against variation in regression parameters. The regression parameter ranges of linear (**a**), quadratic (**b**), and cubic polynomial models (**c**), as well as the quadratic polynomial model with correlated gene pairs (**d**). Overall, 32 genes are adopted from the apoptosis pathway, and 6 pairs of them have relationships of transcription regulation. Thus, there are 33, 65, 97 and 71 parameters in the regression functions of linear, quadratic, cubic polynomial models, and the quadratic polynomial model with correlated gene pairs, respectively

Besides selecting genes based on correlation analysis, we alternatively selected genes that are presented in the apoptosis pathway to predict cell fate. A total of 32 most common genes were used: APAF1, ATF4, BAK1, BAX, BCL2, BCL2L1, BID, CAPN1, CAPN2, CASP8, CASP9, CASP10, CYCS, DAXX, DDIT3, DIABLO, EIF2AK3, EIF2S1, ERN1, FADD, FAS, ITPR1, MAP3K5, MAPK8, MAPK9, MAPK10, MDM2, TNFRSF1A, TRADD, TRAF2, TP53, and XIAP (Fig. 6) [36]. We still applied the linear, quadratic, cubic polynomial models, and quadratic polynomial model with cross terms to refine the cell fate prediction function. 10-fold cross-validation was carried out for each setting. Compared with the correlation based gene selection, here the cross terms were derived from our knowledge of gene regulation. For example, TP53 regulates the transcription of MDM2, then the cross term of TP53×MDM2 was added into the cell fate prediction function. Overall, there are six cross terms. The prediction results are shown in Table 3 and Fig. 7a. The highest accuracy of 84.73% was achieved by the quadratic polynomial model with cross terms. We also analyzed the stability of the models against variation in regression parameters as well as the variances at randomly selected points (Figs. 7b, c and 8). Similarly, 10,000 randomly selected points were used. As the number of genes was fixed, the same set of stability testing points was employed for different degree polynomial models. As shown in Fig. 7b and c, the distribution of variances of the randomly selected points associated with the linear model is very dense, and the values of the variances are much smaller. In Fig. 8, the regression parameters of linear polynomial model are in the smallest fluctuation ranges.

**Table 3:** Cell fate prediction based on different degree polynomials. The genes are selected from the apoptosis pathway

Degree of polynomial	1	2	2 (cross terms)	3
Accuracy	0.8417	0.8463	0.8473	0.8439
MVAV	3.1529e-4	0.0013	0.0022	0.0039

## Discussion

Cell fate decision is very important, as over proliferation or excessive death of cells may lead to various kinds of human diseases. We aim to build models to predict the death probability of a cell based on its gene expression data. A continuous model for steady state data may be suitable for this purpose. A linear model (PLS regression method) was implied in [19, 20] to relate cancer cell phenotypes with the protein activity levels. Although the data were measured at several time points, the linear model can be used to predict phenotypic response at each time point as well. However, a linear model tends to be considered too simple to handle the scenarios of cross talk or feedback in signaling pathways. For the same data set as [20], the authors of [21] proposed an exponential model, which showed better performance in predicting cell death than a linear model. The protein activity levels in [20] were expressed as the fold changes compared with the control samples. Essentially, after a log transformation of the data (protein activity levels), the exponential model becomes a linear one. In practice, a log transformation for the protein activity data was sometimes conducted when performing statistical analysis [37–39]. In our study, we used single-cell gene expression data obtained from [22], in which the RNA-seq method was used to measure the gene expression levels, and the results were reported in reads per kilobase per million mapped reads (RPKM). Typically, a log transformation is necessary for the RPKM data. In one aspect, the gene expression dataset tends to be substantially skewed, but often being log-transformed it can approximate the normal distribution. Moreover, log transformation makes the data more symmetrical, providing much convenience for both direct observation and statistical tests. Thus, the model in [21] is actually linear for our log-transformed gene expression data.

In this work, we intend to find the relationship between single-cell gene expression data and cell fate decision. It is well known that a function can be expressed as a Taylor series under the condition of being infinitely differentiable at a fixed point. We employed this idea and directly used polynomials to represent the cell fate prediction function. The variables of the function are expression levels of specific genes. The genes were selected in two ways, using a correlation based approach and an apoptosis pathway based method. The selected genes could refine the cell fate prediction function in different degree polynomial models (linear, quadratic, cubic polynomial models, and quadratic polynomial model with cross terms of correlated gene pairs). 10-fold cross-validation was carried out for model validation. In addition, we analyzed the stability of each model by evaluating the ranges of the parameters, as well as assessing the variances of the predicted values at some randomly selected points. In the scenario of gene selection based on correlation analysis, the prediction accuracies (about 84%~86%) of different degree polynomial models do not show much difference. However, linear regression model performed the best in the stability analysis when the same number of genes was used to refine the prediction functions (Tables 1 and 2). Take the number of 10 applied genes as an example (Figs. 4 and 5). The parameters of the linear regression model vary in a smaller scale compared with those of other degree polynomial regression models. In addition, the PDFs of different models show that the 10,000 variances (corresponding to a total of 10,000 tested points) of the linear model are much smaller and the distribution is very dense. The same situation occurs when genes are selected from the apoptosis pathway. Then we compared the performance of the models based on the results of the two gene selection methods. Although the prediction accuracy of the model based on correlation analysis outcomes can achieve 86.62%, higher than the best accuracy of the pathway based method, the models composed of genes from the apoptosis pathway are more stable (comparison of the same degree polynomial models, Tables 1~3). The difference in the stability may be caused by the genes used in regression. In each training process (to learn the cell fate prediction function), the involved genes may be slightly different if the correlation based method is used, but the same set of genes is employed when using the apoptosis pathway based approach.

As discussed above, the prediction accuracies of the polynomial model of different degrees show little difference, but the linear polynomial model performed the best in terms of stability. To further analyze the behavior of a biochemical system, it is desirable to explore the theoretical description of the chemical reactions [40–43].

## Conclusions

In this study, we proposed a polynomial based model to predict cell fate. To refine the prediction function, genes were selected by using a correlation analysis approach as well as an apoptosis pathway based method. We employed different degree polynomials to refine the cell fate prediction function from single-cell gene expression data of human pancreatic cells. Using the two gene selection methods, the prediction accuracies of different degree polynomial models are very close, but the linear regression model performs much more stable than others. When comparing the performance of linear regression model based on the results from the two gene selection methods, the prediction accuracy of the model with correlation analysis outcomes is a little higher (86.62% vs. 84.17%) than that of the model based on genes from the apoptosis pathway. However, the model with genes from the apoptosis pathway is more stable (3.1529e-4 vs. 7.7577e-4, MVAV). Thus, it is promising to use linear model to associate cell fate decision with gene expression data for the pancreatic cells. In addition, the genes in a specific pathway are preferred to conduct the regression process. This linear model could be extended to the cell fate prediction of other cells, and thereby facilitate research on human diseases caused by cell fate dysregulation.

## Abbreviations

CDF: Cumulative density function
MVAV: Mean value of all the variances
PDF: Probability density function
PLS: Partial least squares
RPKM: Reads per kilobase per million mapped reads
T2D: Type 2 diabetes
